# Addressing Access to Child Mental Health Services in Primary Care: Implementation and Feasibility of the Colorado Pediatric Psychiatry Consultation and Access Program

**DOI:** 10.3390/children12111425

**Published:** 2025-10-22

**Authors:** Kaitlin A. Whelan, J. Kyle Haws, Susan Young, Ryan Asherin, David Keller, Sandra Fritsch

**Affiliations:** 1 Department of Pediatrics, University of Colorado School of Medicine, Aurora, CO 80045, USA; david.keller@childrenscolorado.org; 2 Department of Psychiatry, University of Colorado School of Medicine, Aurora, CO 80045, USAsandra.fritsch@childrenscolorado.org (S.F.); 3 Adult & Child Center for Outcomes Research & Delivery Science, University of Colorado School of Medicine, Aurora, CO 80045, USA; 4 Pediatric Mental Health Institute, Children’s Hospital Colorado, Aurora, CO 80045, USA

**Keywords:** pediatric mental health, primary care, consultation services, child psychiatry access programs

## Abstract

Background/Objectives: Pediatric mental health is a major public health concern worldwide and primary care providers struggle to meet the growing demand for mental healthcare. Child Psychiatry Access Programs have emerged to fill gaps in primary care provider (PCP) training, confidence, and workflow support. This study aimed to describe the iterative development of a Child Psychiatry Access Program and present initial findings on its reach and feasibility in supporting PCPs. Methods: The Practical, Robust Implementation and Sustainability Model (PRISM) implementation framework guided the development and evaluation of the program. Pre-implementation surveys and invested partner interviews informed the creation of a multidisciplinary program comprising three components: (1) consultation services and resource navigation, (2) education and training, and (3) provider care guides. The program was then implemented, and reach was assessed via consultation calls, attendance at education and training series, resource navigation encounters, and care guide usage. Feasibility was evaluated through pre- and post-series self-reported ratings across six learning objectives. Results: Pre-implementation evaluation indicated high provider interest across all educational modalities. The resulting program included consultation services, education and training, resource navigation, and provider care guides. Educational trainings led to significant improvements in self-reported knowledge and confidence across six learning objectives, including assessment, treatment planning, family engagement, and navigating local resources. Resource navigation primarily facilitated ongoing management within the primary care setting, with PCPs retaining care in the majority of cases. Engagement with the Colorado Care Guide demonstrated sustained reach, with over 4600 page views from 1300 active users, reflecting broad and ongoing utilization of program resources. Consultation call data mirrored these trends, highlighting both frequently addressed diagnoses and expanding program reach over time. Conclusions: Child psychiatry access programs help support access to youth mental health care in the primary care space and offer potential solutions to workforce limitations during an era of increasing mental health concerns in youth and teens. Findings from this implementation may inform adaptation of child psychiatry access programs in other regions seeking to expand mental health support for children and adolescents in primary care settings.

## 1. Introduction

Pediatric mental health has emerged as a major public health concern worldwide, with rates of anxiety, depression, behavioral disorders, and suicidality rising significantly over the past two decades [1,2,3,4]. Despite increased awareness, many children and adolescents lack timely evaluation and access to mental healthcare [5]. Barriers to access include a shortage of child and adolescent mental healthcare professionals, fragmented care systems, stigma, costs, and geographic disparities in specialist availability [6,7,8,9]. As a result, primary care providers (PCPs) often serve as the first, and sometimes only, point of contact for families [10,11]. While they serve as the primary mental health contact, PCPs receive limited training in managing psychiatric conditions, and they fulfill this role because their patients often have nowhere else to turn [12]. Thus, it is necessary to develop programs to support PCPs and their patients in settings where they seek care.

While there is growing recognition of the importance of primary care in managing pediatric mental health, significant challenges persist [13,14]. These challenges include a lack of formal residency training in pediatric mental health, a limited understanding of available community mental health resources, and a lack of knowledge about screening instruments [15,16,17]. In a study evaluating barriers to providing mental health assessment and treatment, PCPs report that they have inadequate training and a lack of confidence in addressing pediatric mental health problems [14]. Beyond inadequate training, PCPs often report structural barriers, including insufficient time, both to pursue training and to coordinate mental health treatment plans during visits [14,18]. While these barriers persist, there is a need for improved pediatric mental health training and support for primary care practices [12].

To support primary care practices, programs have been created and implemented to deliver mental health screening and treatment for youth within the primary care medical home [19,20,21,22]. In 2004, for example, a Child Psychiatry Access Program (CPAP) was established in Massachusetts to provide mental health consultation and support within the primary care workflow [21,22]. In these consultation programs, PCPs consult with a child psychiatrist for diagnostic clarifications or medication management. Evidence suggests that these consultation models improve provider confidence and knowledge in medication management, screening for mental health issues, and even improve patient outcomes [20,21,23].

Despite these promising results, existing programs face ongoing challenges, including variable reach across geographic areas, inconsistent integration within primary care workflows, and limited data on implementation outcomes and long-term impacts on provider confidence and patient health. To address these gaps, we developed a multi-component program that integrates consultation services and resource navigation, education and training, and online care guides. This paper describes the pre-implementation assessment, program development, and initial evaluation, focusing on program reach and feasibility in improving PCP knowledge, confidence, and comfort in managing pediatric mental health.

## 2. Materials and Methods

### 2.1. Implementation Science Framework

**Figure 1:**
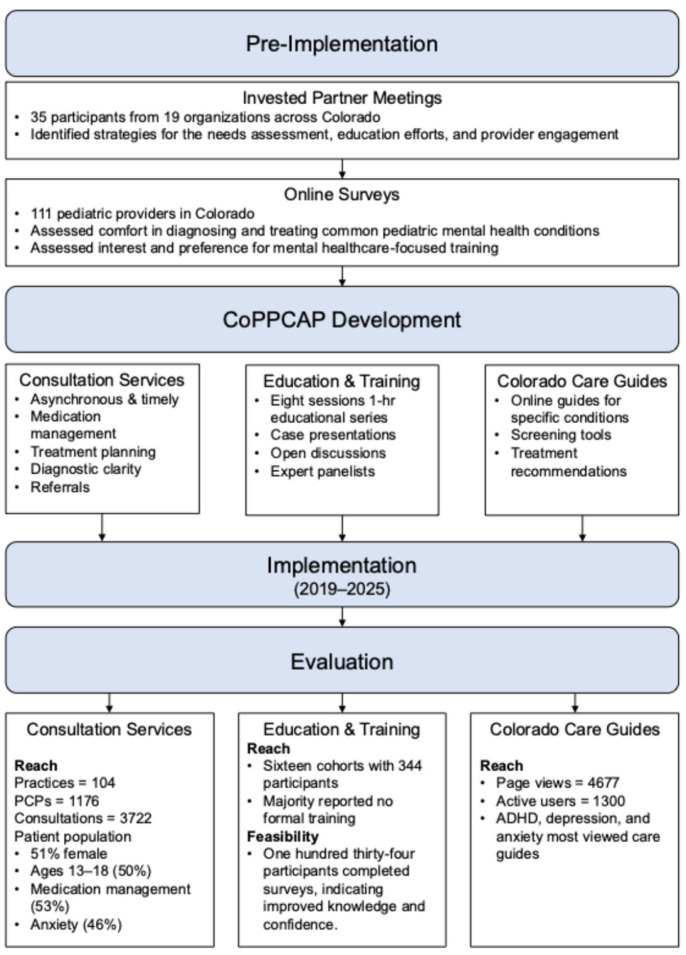
Project Flow from Needs Assessment, Program Development, Implementation, and Evaluation.

The Practical, Robust Implementation and Sustainability Model (PRISM) guided the development and evaluation of the program [24], which provides a structured approach to understanding the multilevel context of pediatric primary care, assessing the needs of providers and practices, and evaluating early implementation outcomes. The pre-implementation phase involved assessing provider needs through small groups and surveys to identify barriers, facilitators, and priorities for managing pediatric mental health concerns. These insights informed the iterative development of the program. PRISM emphasizes the role of contextual factors in influencing implementation success. This includes understanding providers’ knowledge, confidence, and comfort in managing pediatric behavioral health, how external factors such as available resources, practice infrastructure, and broader system-level influences affect engagement, and how the design and delivery of program components interact with both those delivering and receiving the intervention. Consideration of these elements guided the evaluation of key implementation outcomes, including reach across all program components (e.g., number of providers and practices engaged, geographic spread, and program utilization) and feasibility of the educational components (changes in provider knowledge, confidence, satisfaction, and comfort). This approach ensured that program development and evaluation were grounded in an understanding of the system, provider, and patient contexts that shape successful implementation, summarized in Figure 1.

### 2.2. Key Invested Partner Interviews and Online Surveys

The initial step in the pre-implementation evaluation was to hold an invested partner summit with participants recruited from community mental health organizations, the Colorado Department of Health Care & Financing, Children’s Hospital Colorado, and Colorado’s Office of Behavioral Health. Participants represented different state departments, school-based staff, community organizations, parent organizations, and other access programs. The group met to discuss the existing mental health landscape in Colorado. Each participant joined two small groups covering three topics: *Needs Assessment & Evaluation*, *Education*, and *Provider Engagement*. Each group was then led through a series of questions. For the *Provider Engagement* topic, the questions focused on identifying ways to successfully engage providers across the state and integrate the consultation model with practice workflows. During the *Needs Assessment & Evaluation* topic, participants were asked to identify communities and practices that would benefit from a consultation program and identify strategies for assessing their perspectives. In the *Education* topic, participants were asked to identify their training needs, types of learning venues and styles, the value of electronic resources, areas of competency, and the content of training and consultation. Results from the invested partner meeting informed the development and dissemination of the online survey.

Guided by the results from the invested partner meetings, an online survey was sent to primary care providers across Colorado to understand the current primary care context and identify the needs of PCPs in addressing pediatric mental health. The surveys examined practice characteristics to ensure a broad representation of settings, providers’ comfort diagnosing and treating common mental health conditions, perceptions of barriers to care, and evaluated interest in different formats of engaging with the consultation program, which included different educational offerings and consultations. Surveys were collected between 1 May 2019 and 24 July 2019. Outreach support was provided by the Colorado Chapter of the American Academy of Pediatrics (AAP), the Physician Relations Department of Children’s Hospital Colorado, and the Colorado State Innovation Model team.

### 2.3. Developing the Colorado Pediatric Psychiatry Consultation and Access Program (CoPPCAP) Team

We assembled a multidisciplinary team, including a care coordination navigator, child and adolescent psychiatrists, a medical director, program manager, data manager, program evaluator, and pediatric primary care consultants. The care coordinator navigator serves as the primary point of contact for PCPs, collecting information on patient needs and requested services, and arranging consultations with psychiatrists. Child and adolescent psychiatrists provide consultations and are involved in leading educational offerings. The medical director and project director provide project oversight, including strategic planning and visioning, project management, budget oversight, and oversight of future implementation strategies of the project. A program evaluator works closely with the data manager and clinical care coordinator to ensure that the necessary data are collected on the providers and patients served by the project and analyzes the data to evaluate whether services are meeting project performance goals. Pediatricians serve as primary care consultants, providing input and helping to develop initiatives. Members from the multidisciplinary team met weekly to ensure the program is meeting its aims as well as to continue program innovations.

### 2.4. Enrollment with CoPPCAP

CoPPCAP partners with pediatric and family medicine practices, as well as school-based health centers across Colorado, including those serving rural and historically underserved populations. Practices provide basic information about their clinic and patient population upon enrollment, but access to CoPPCAP consultation calls and online resources is open to all providers serving pediatric patients. Participation at any level is free and payor-blind, meaning that access is not restricted by patient insurance type or funding source.

### 2.5. Implementation Evaluation and Data Analysis Plan

To evaluate early implementation outcomes, the program examined two primary data sources: consultation call records and provider surveys. Consultation calls capture detailed information on each encounter, including provider and practice characteristics, patient demographics, clinical history, family history, current presentation, and the support or questions requested by the primary care provider. Child and adolescent psychiatrists document recommendations (e.g., medication changes) and referrals to specialists or community resources. All consultation data are recorded in a REDCap database. Provider surveys assess satisfaction with program offerings, perceived impact on clinical care, and confidence in addressing pediatric behavioral health concerns. Surveys are conducted semiannually for consultation services and immediately following educational sessions to evaluate curriculum relevance, quality of delivery, impact on skills and competence, and self-reported confidence. Participation is voluntary and anonymized. Usage data for Care Guides, including page views and active users, are also collected to inform program reach.

The program evaluated two key outcomes: (1) reach and (2) feasibility. Reach was assessed across all program components using metrics such as the number of participating providers, practices, and patients; geographic distribution; and engagement patterns. Descriptive statistics (counts, percentages, and frequencies) summarized these indicators to capture both the breadth and scope of program engagement. Feasibility and acceptability were assessed for the educational program, focusing on changes in provider knowledge, confidence, comfort, and satisfaction. Pre- and post-series ratings of learning objectives and self-reported comfort were compared using paired-sample *t*-tests for each objective, with a significance threshold of *p* ≤ 0.001 to account for multiple comparisons.

## 3. Results

### 3.1. Pre-Implementation Evaluation

#### 3.1.1. Invested Partner Meetings

Thirty-five participants representing nineteen different organizations or agencies attended the invested partner meetings on 11 January 2019. When examining the *Needs Assessment* groups, key partners recognized the importance of conducting needs assessments among providers, practices, and communities. They recommended supplementing with direct input from families, schools, and federally qualified health centers. This multi-layered approach was developed to ensure that program priorities reflect real-world challenges and community context rather than payer-driven constraints. Evaluation strategies were developed to capture not only program reach but also changes in provider confidence, workflows, and capacity to deliver mental health care.

In the *Education* groups, emphasis on educational efforts for both child psychiatrists and PCPs was developed to include adult learning theory, interactive, and case-based formats. A mix of strategies, including traditional lunch-and-learns, virtual sessions, and collaborative learning models, was deemed necessary to meet providers where they are and sustain engagement across both urban and rural practices. Invested partners also identified workflow design, practice transformation, and provider resiliency, with continuing education credits (CME/MOC) and peer-to-peer learning. Building in ongoing support mechanisms, such as office hours, hotlines, or regional hubs, was considered critical for implementation and sustainability. It was also recommended to include parents in certain educational sessions to serve as panelists with lived experience.

For the *Provider Engagement* groups, successful provider engagement was described as requiring relationship-building, clarity about program services, and attention to practice workflows. Invested partners emphasized the importance of in-person outreach and local champions in building trust, particularly in rural areas, while supplementing these efforts with virtual strategies for sustainability. The invested partners highlighted the importance of engaging the entire care teams so that workflows and technology adoption are feasible and sustainable. Providers expressed the need for reassurance that CPAPs would support, rather than replace, their role, and that recommendations would be practical within their scope. Partners also identified effective strategies for sustaining engagement through incentives such as CME/MOC credit, peer support networks, and integration with existing meetings or transformation initiatives.

#### 3.1.2. Online Surveys

Following the invested partner summit, surveys were collected from 111 pediatric providers in Colorado. When examining the practice settings of the responding providers, over 75% were in a large (8 or more physicians or advanced practice nurses) or medium-sized (3–7 physicians or advanced practice nurses) practice. Most providers practiced in private settings (63.2%), followed by those in hospital-based clinics (17.1%), integrated care clinics (7.9%), school-based primary care settings (5.3%), Federally Qualified Health Centers (2.6%), and other specialty settings (1.3%). The majority of respondents (85%) were physicians, with the remainder nearly evenly distributed among nurse practitioners, physician assistants, and practice managers. One-third of providers reported that at least half of their patients were publicly insured, while another 43.8% indicated that fewer than one-quarter of their patients received public insurance benefits.

The survey assessed the comfort level of primary care providers in diagnosing and treating common mental health conditions. As shown in Table 1, more than 80% of provider respondents felt at least somewhat comfortable in diagnosing ADHD, depression, anxiety, and developmental delays. In contrast, substantially fewer providers reported comfort in diagnosing substance use disorders. Providers were also evaluated on their self-reported comfort in managing these same mental health conditions. Overall, comfort levels were lower across all conditions, with the least confidence reported in managing substance use disorders.

To gauge participants’ interest and preference for mental healthcare-focused trainings, participants were asked, “How interested are you in becoming more skilled or comfortable with managing your patients’ mental health needs within your clinic?” Almost all replied, “very interested” or “somewhat interested,” underscoring the widespread interest for additional training and support. When asked about preferred formats, participants did not show a clear preference for one type of educational or consultation opportunity over another. Instead, nearly 90–95% expressed interest across all modalities, which included live video group learning with child psychiatrists, topical “Lunch and Learn” sessions, same-day phone consultations, asynchronous digital consultations, and one-time face-to-face consultations. These findings suggest that providers are interested to engage in mental health skill-building and value a range of accessible, flexible training and consultation options.

The survey assessed providers’ perceptions of the barriers patients and families face when accessing mental health care. The most frequently reported barrier was insurance coverage being too narrow, with nearly two-thirds of respondents expressing this concern. A similar number responded that the waitlist for appointments was too long. A little over a third reported their patients and families did not have access to the specialty mental health care they needed. Sociodemographic factors were also commonly cited as barriers to access, including out-of-pocket costs, excessive travel distance to the provider, and inconvenient available appointment times.

### 3.2. Iterative Program Development

Using information from the invested partner meeting and the surveys, the program developed the core services, including consultation services and resource navigation, education and training, and online care guides.

#### 3.2.1. Consultation Services and Resource Navigation

Based on participant feedback, the program developed a consultation services pathway, which is an asynchronous and timely psychiatric consultation available to PCPs via phone or secure electronic health platforms. A primary care provider will call or message about a specific patient. The care coordination navigator will take the call and notify the on-call psychiatrist of the consult. Consultations cover diagnostic questions, treatment planning, medication management, and referral guidance, and are returned within 30 to 45 min or at a preferred time of the consulting PCP.

In addition to consultation services, the care coordination navigators assist providers in identifying community-based mental health services and supports. The resources are tailored to the specific patient’s needs and insurance requirements for the required diagnosis, offering evidence-based care.

#### 3.2.2. Education and Training

A seven- to eight-session series of one-hour educational modules focused on managing mental health concerns in the primary care setting was developed and delivered through the ECHO Colorado platform. The series titled “*Pediatric Psychiatry in Primary Care: Core Essentials*” was designed to provide core knowledge and skills for treating mental health challenges in primary care settings. The series’ modules consist of 20–30-min lectures and 20–40-min group discussion periods, allowing for case presentations, open discussion, and professional peer support. Child and adolescent psychiatrists, as well as the pediatrician consultants, present the lectures, while expert panelists are available for the group discussion period. Expert panelists include child psychiatrists, child psychologists, pediatricians, pediatric psychiatric pharmacists, and trained parent peer support specialists with lived experience. Topics in the *Core Essential* series cover screening and assessment, what therapy is, and addressing mental health concerns in the primary care setting.

In addition to the *Core Essentials* series, two advanced courses covering more complex disorders, such as disruptive behavior problems, substance use issues, and how to assess and manage comorbidity, were later developed. After recognizing an increase in burnout among provider participants, the series also incorporated a self-care component into each educational session, where participants took a few moments to reflect on symptoms of burnout and how to make actionable changes. Other educational offerings have included annual learning collaboratives focusing on four specific learning areas, presentations at regional professional meetings, and requests from practices for specific learning topics.

#### 3.2.3. Colorado Care Guides

Recognizing that providers and practices have different levels of comfort managing mental health concerns and learning styles, the program developed the Colorado Care Guides around specific conditions. Available online, the Colorado Care Guides have been created on high-yield topics, including assessment and screening (including free and publicly/downloadable screening tools in multiple languages), providing the basics of evaluation, diagnosis, and treatment for conditions including ADHD, anxiety, depression, disruptive behaviors and suicide.

### 3.3. Implementation Evaluation

#### 3.3.1. Consultation Services and Resource Navigation

Between 2019 and 2025, CoPPCAP enrolled 104 primary care practices, comprising 1176 primary care providers, in its program. The PCPs enrolled provide care for 732,111 patients in Colorado. The program has received 3722 patient consultation questions from primary care providers. CoPPCAP consultations span a broad geographic footprint, with services accessed from a diverse array of counties across Colorado. Consultation length averaged 14.5 min, with the most frequent durations being 15 min (30%), 10 min (28%), and 20 min (16%). There has been a steady increase in consultation volume from late 2019 to mid-2025.

Consultations were most frequently initiated by pediatric practices (80%), followed by family practices (11%), school-based health centers (4%), and combined pediatric/family practices (4%). The consultation calls served a diverse patient population, with a relatively balanced gender distribution: 51% female, 46% male, and 1% non-binary. The majority of consultation calls focused on adolescents aged 13–18 (50%), followed by children aged 7–12 (32%), those in early childhood (0–6 years, 11%), and young adults aged 18 years and older (6%). The most frequently supported diagnoses were anxiety (46%), depression (37%), and ADHD (33%), followed by autism (14%), PTSD (7%), and oppositional defiant disorder (7%). Multiple diagnoses and outcomes could be coded for each call. Diagnosis patterns varied by age group, with ADHD most prevalent among children aged 7–12, and anxiety and depression most common among adolescents aged 13–18.

During consultation calls, medication management emerged as the predominant reason for consultation, with 53% of calls related to ongoing medication management and 29% concerning medication initiation. Other common reasons included psychological and behavioral health concerns (24%), community resource assistance (14%), and diagnostic clarification (5%). If the outcome of the call includes resource needs, the care navigators found resources.

**Figure 2:**
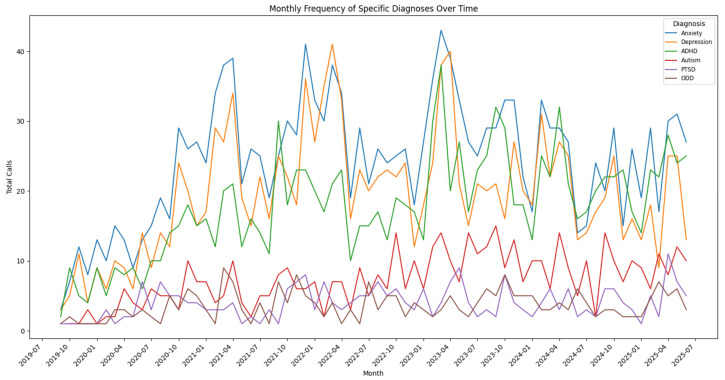
Trends in Consultations Call Frequency by Most Common Diagnosis Over Time.

Beyond medication management and diagnostic clarification, other outcomes included clarifying differential diagnosis (36%) and referrals to therapists (35%). Less common but clinically significant outcomes included referrals to psychiatry (9%), and neuropsychology (3%). A smaller proportion of cases involved higher-intensity recommendations, such as partial hospitalization or intensive outpatient programs (5%), crisis evaluation or ED follow-up (<1%), or inpatient care (<1%). Overall, these findings, summarized in Figure 2, underscore CoPPCAP’s strong emphasis on supporting ongoing care within the primary care setting, while also facilitating access to higher levels of care and specialized services as needed.

#### 3.3.2. Education and Training

Sixteen cohorts of the eight-session *Core Essentials* series have been completed, enrolling a total of 344 participants. Among those with available registration data, 98% reported working directly with patients, 89% practiced in primary care settings, 85% in settings accepting public insurance, and 54% identified as practicing in underserved environments. Healthcare providers comprised 89% of participants, followed by behavioral health providers (8%), practice management and health education professionals (1%), and participants from other professions (2%). Most participants are based in Colorado (88%), with 21% of them from rural areas. A majority of participants reported they had minimal or no formal training prior to the series in addressing behavioral health concerns in primary care patients.

A total of 134 anonymous post-course surveys were completed. Participants reported high levels of satisfaction with the series, with 100% of respondents agreeing or strongly agreeing with all seven satisfaction questions (e.g., “*This series makes me better at my job*,” “*I was satisfied with this series overall*”). Participants were also asked to rate their comfort addressing behavioral health concerns in their clinical settings before and after the lecture series. At baseline, only 1% of participants reported feeling very comfortable. Following the series, 23% of participants reported being very comfortable, while only 11% continued to feel more uncomfortable than comfortable. These findings suggest a substantial increase in providers’ confidence in addressing behavioral health needs within their practice.

In addition to improving providers’ confidence, significant improvements were observed across all learning objectives when comparing pre- and post-series ratings. Paired-sample *t*-tests demonstrated improvements in participants’ self-rated knowledge and confidence, with all comparisons reaching statistical significance (*p* < 0.001). Specifically, participants reported increased ability to describe the different presentations of behavioral health conditions (*M*_pre_ = 3.03, *M*_post_ = 3.74, *T* (230) = −18.83, *p* < 0.001), summarize common assessment issues (*M*_pre_ = 2.88, *M*_post_ = 3.69, *T* (230) = −22.69, *p* < 0.001), summarize treatment issues (*M*_pre_ = 2.86, *M*_post_ = 3.68, *T* (230) = −21.30, *p* < 0.001), discuss the family perspective on assessment (*M*_pre_ = 2.72, *M*_post_ = 3.56, *T* (230) = −18.69, *p* < 0.001) and treatment (*M*_pre_ = 2.68, *M*_post_ = 3.48, *T* (230) = −17.82, *p* < 0.001), and serve as a local resource in their workplace or community (*M*_pre_ = 2.68, *M*_post_ = 3.62, *T* (230) = −16.95, *p* < 0.001). These findings, summarized in Table 2, highlight both the educational feasibility of the ECHO series and its impact on participants’ confidence in applying behavioral health knowledge to practice.

#### 3.3.3. Care Guides

Since the launch of the first Care Guide on 14 January 2021, through 30 June 2025, the Colorado Care Guide webpage has received 4677 page views from 1300 active users. These data suggest consistent engagement with the resource over time and indicate a meaningful level of reach within the intended audience. Tracking functionality to look at which care guide was viewed was only enabled within the past year (2025). Since tracking was started, the ADHD care guide was viewed and downloaded most (32%), followed by the depression care guide (22%) and anxiety care guide (21%).

## 4. Discussion

We conducted a pre-implementation evaluation using invested partner meetings and online surveys, iteratively developed a program tailored to the primary care context, and assessed early implementation outcomes. The pre-implementation evaluation revealed a strong interest among providers in providing mental healthcare, but also identified limitations in their comfort levels, especially for substance use. We developed a program tailored to the primary care context and provider needs by offering consultation calls, education and training, resource navigation, and care guides. After developing the program, we implemented it from 2019 to 2025, finding that it further enhances provider knowledge, confidence, and ability to act, offering tailored guidance for addressing common pediatric mental health concerns and overcoming system-level barriers, which is consistent with similar programs [20,23]. Overall, our findings demonstrate the feasibility and potential impact of a multi-component consultation model to strengthen pediatric mental health care within primary care.

A key component of implementation success relies on a rigorous pre-implementation evaluation [25,26]. Our pre-implementation assessment underscored the importance of grounding program development in the real-world context of primary care. Building on the principles of community engagement [27], a wide degree of representativeness among invested partners ensured that the program effectively addressed community needs. During these invested partner meetings, education emerged as a critical component, with recommendations for interactive, case-based formats, adult learning strategies, and multiple delivery modalities, including virtual sessions, lunch-and-learns, and collaborative learning, to engage providers across both urban and rural settings. Education and ongoing training have emerged as important components to support PCPs in delivering mental health services [28].

In addition to providing education and training opportunities, participants emphasized the importance of integrating workflow design, practice transformation, and provider resiliency into educational programming, with ongoing support mechanisms such as office hours, hotlines, and regional hubs, as well as inclusion of parents as panelists to provide lived-experience perspectives, which has been supported in other research [28]. Other components of effective provider engagement include relationship-building, clear communication about program services, and attention to team workflows. In-person outreach and local champions are vital in rural areas.

Building on the results from the invested partner meetings, the needs assessment revealed that primary care providers generally reported high levels of comfort screening for and diagnosing most pediatric mental health conditions except substance use, which is consistent with previous research [29]. PCPs report significant barriers towards screening for substance use, including a lack of treatment options, a lack of substance use providers, and a lack of information on treatment options [29].

While participants reported high levels of comfort screening for mental health conditions, they also reported lower levels of comfort treating mental health conditions. In addition, during structured educational offerings, self-reported comfort across both diagnosis and treatment domains was lower overall. Importantly, what providers mean by “comfort with treatment” remains unclear. Future work should explore whether this comfort reflects primarily the use of medication management, referral practices, or openness to delivering brief, evidence-based behavioral interventions within primary care. Clarifying providers’ interest and capacity in delivering effective interventions will be essential for tailoring education, consultation, and implementation supports that build both diagnostic accuracy and confidence. Overall, findings from the pre-implementation evaluation and needs assessment informed the iterative development of a program designed to enhance provider capacity, confidence, and workflow feasibility in delivering pediatric mental health care.

After completing the pre-implementation evaluation, we iteratively developed a program designed to support PCPs in delivering pediatric mental health care. Consultation services provided timely access to psychiatric expertise for diagnostic, treatment, and referral questions, extending specialist support to providers across both urban and rural settings. The education and training series combined brief lectures and interactive group discussions to build knowledge, confidence, and peer support, directly targeting measurable improvements in provider competence and comfort. Resource navigation connected PCPs with community-based, evidence-informed services tailored to patient needs, while the Colorado Care Guides offered accessible, condition-specific guidance for common behavioral health concerns.

Once we developed the program, we opened enrollment and implemented the program across Colorado to address gaps in pediatric mental healthcare by providing free and timely psychiatric consultation and supporting primary care providers in navigating local resources. With patients served from 50 of Colorado’s 64 counties, CoPPCAP has successfully extended reach to areas with historically limited access, ensuring that care is delivered within the communities where children and families live. Trends in call type and content further underscore the program’s impact. Consultation volume has steadily increased since the program’s launch in 2019, reflecting both the growing awareness among providers and the persistent need for timely mental health support in pediatric primary care. Although, as noted by others, the call volumes may not drop over time but instead become more complex [22]. Future work should investigate the effectiveness of consultation calls within the clinical workflow and identify any potential disruptions.

Consistent with other CPAPs [22], diagnostic patterns were developmentally consistent, with ADHD most prevalent in school-aged children and anxiety and depression most common in adolescents. Outcomes of consultation calls mirrored these trends, with general medical education and continued PCP management being the most frequent results, followed by medication adjustments, diagnostic clarification, and referral to therapy. At the same time, the high proportion of medication-related calls highlights an opportunity to expand the scope of consultation. Future iterations of the program might integrate structured coaching for primary care providers in brief, evidence-based behavioral interventions (e.g., behavioral activation for depression, interoceptive exposures for panic). This type of support could complement medication management and further build primary care capacity to address pediatric mental health comprehensively.

Another important aspect of our program was to increase knowledge and comfort in managing pediatric mental health concerns in primary care. As such, we developed the *Core Essentials* series, which effectively improved self-reported knowledge and confidence in managing pediatric behavioral health concerns. Consistent with previous consultation and education models [23], participation in the series resulted in substantial gains, with the proportion of providers feeling very comfortable managing mental health in primary care. Statistically significant improvements were observed across all learning objectives, including assessment, treatment planning, family engagement, and functioning as a local resource. While these findings highlight the series’ educational feasibility, it is important to note that they reflect self-reported confidence and skill, and further research is needed to determine whether these gains translate into observable changes in clinical practice and patient outcomes.

Building on the foundation of consultation services and educational offerings, the Colorado Care Guides further extended CoPPCAP’s reach by providing PCPs with actionable tools and guidance. The Care Guides demonstrated consistent engagement, with over 4600 page views from 1300 active users, indicating a meaningful reach. ADHD was both the most common diagnosis prompting consult calls and the most frequently downloaded care guide. Depression and anxiety were also frequent reasons for consultation, with corresponding care guides ranking next in download frequency. However, patterns of use, including the distinction between unique and repeat users, geographic distribution, and trends over time, remain unclear. More granular research is needed to understand how providers are incorporating these resources into practice and to optimize these components alongside consultation and educational offerings.

Limitations of this study and program include ongoing challenges in ensuring equitable geographic distribution of services and sustaining funding beyond federal grant cycles. Only 134 of 344 participants completed the end-of-series surveys for the educational and training program, which may limit the representativeness of the findings and introduce potential response bias. Self-reported measures may not fully capture practice change. As a result, one future direction may be surveying the participants at an additional interval to see if comfort and knowledge levels remain higher than prior to the educational series. During consultation calls, because patient information is deidentified, the program cannot directly assess patient-level outcomes or link provider participation to changes in clinical care. Utilization patterns may change over time, as providers may rely less on consultation services once they become more comfortable managing mental health concerns. Another important consideration is reach. While CoPPCAP has engaged a wide range of providers, practices, and regions, the program likely is missing key perspectives. Understanding which providers and settings are not participating and, by extension, which patients are not benefiting, remains critical for ensuring equitable access and health equity [24]. The current evaluation is also largely cross-sectional, and future efforts will require longitudinal follow-up to examine the durability of changes in provider knowledge, confidence, and practice behaviors, ideally using more rigorous psychometric measures. Further research is needed to assess the program’s impact on patient outcomes, including clinical improvements, access to care, and reductions in disparities.

## 5. Conclusions

The findings underscore the promise of integrated consultation models in supporting primary care capacity to address pediatric mental health needs. Child Psychiatry Access Programs, such as CoPPCAP, offer a comprehensive approach to supporting providers through consultation, education, and resource navigation, thereby helping to overcome workforce limitations and enhance access to quality care. As youth mental health needs continue to rise, similar models could be adapted in other regions to meet growing demand. CoPPCAP has become a vital resource for healthcare providers across Colorado, providing real-time psychiatric expertise that enhances clinical decision-making, supports mental health assessments, and improves care delivery in both urban and rural settings.

## Figures and Tables

**Table 1:** Provider Comfort with Diagnosing and Treating Common Pediatric Mental Health Conditions.

Condition	Comfortable Diagnosing	Uncomfortable Diagnosing	Comfortable Treating	Uncomfortable Treating
ADHD	86%	14%	88%	12%
Depression	88%	12%	75%	25%
Anxiety	91%	9%	74%	26%
Developmental Delays	92%	7%	79%	22%
Substance Use	59%	41%	23%	77%

**Table 2:** Improvement in Learning Objectives Following Educational Training.

Learning Objectives	Pre-ECHO (Mean)	Post-ECHO (Mean)	*t*-Value	df	*p*-Value
How would you rate your ability to describe the different presentations of the behavioral health conditions covered in this series.	3.03	3.74	−18.825	230	<0.001
How would you rate your ability to summarize the common assessment issues of the behavioral health conditions covered in this series.	2.88	3.69	−22.692	230	<0.001
How would you rate your ability to summarize the common treatment issues of the behavioral health conditions covered in this series.	2.86	3.68	−21.304	230	<0.001
How would you rate your ability to discuss the family perspective of the assessment of the behavioral health conditions covered in this series.	2.72	3.56	−18.690	230	<0.001
How would you rate your ability to discuss the family perspective of the treatment of the behavioral health conditions covered in this series.	2.68	3.48	−17.822	230	<0.001
How would you rate your ability to serve as a local resource within your workplace or community for the topic of this ECHO series.	2.68	3.62	−16.952	230	<0.001

## Data Availability

Dataset available on request from the authors. The raw data supporting the conclusions of this article will be made available by the authors on request.

## Abbreviations

The following abbreviations are used in this manuscript:

PCP	primary care providers
PRISM	Practical, Robust and Sustainability Model
CPAP	Child Psychiatry Access Program
AAP	American Academy of Pediatrics
CoPPCAP	Colorado Pediatric Psychiatry Consultation and Access Program
CME/MOC	continuing medical education
ADHD	attention deficit hyperactivity disorder
PTSD	post-traumatic stress disorder
ED	emergency department
